# Gut Microbiota-Dependent Marker TMAO in Promoting Cardiovascular Disease: Inflammation Mechanism, Clinical Prognostic, and Potential as a Therapeutic Target

**DOI:** 10.3389/fphar.2019.01360

**Published:** 2019-11-19

**Authors:** Shengjie Yang, Xinye Li, Fan Yang, Ran Zhao, Xiandu Pan, Jiaqi Liang, Li Tian, Xiaoya Li, Longtao Liu, Yanwei Xing, Min Wu

**Affiliations:** ^1^Guang’an men Hospital, China Academy of Chinese Medical Sciences, Beijing, China; ^2^Beijing University of Chinese Medicine, Beijing, China; ^3^Department of Cardiovascular, Beijing Longfu Hospital, Beijing, China; ^4^Xiyuan Hospital, China Academy of Chinese Medical Sciences, Beijing, China

**Keywords:** trimethylamine N-oxide, atherosclerosis, cardiovascular disease, inflammation mechanism, clinical prognostic stratification, therapy

## Abstract

Cardiovascular disease (CVD) is the leading cause of death worldwide, especially in developed countries, and atherosclerosis (AS) is the common pathological basis of many cardiovascular diseases (CVDs) such as coronary heart disease (CHD). The role of the gut microbiota in AS has begun to be appreciated in recent years. Trimethylamine N-oxide (TMAO), an important gut microbe-dependent metabolite, is generated from dietary choline, betaine, and L-carnitine. Multiple studies have suggested a correlation between plasma TMAO levels and the risk of AS. However, the mechanism underlying this relationship is still unclear. In this review, we discuss the TMAO-involved mechanisms of atherosclerotic CVD from the perspective of inflammation, inflammation-related immunity, cholesterol metabolism, and atherothrombosis. We also summarize available clinical studies on the role of TMAO in predicting prognostic outcomes, including major adverse cardiovascular events (MACE), in patients presenting with AS. Finally, since TMAO may be a novel therapeutic target for AS, several therapeutic strategies including drugs, dietary, etc. to lower TMAO levels that are currently being explored are also discussed.

## Introduction

Atherosclerosis (AS) and resulting cardiovascular diseases (CVDs) are serious threats to human health (Anderson et al., 1991; Vilahur et al., 2014). Multiple studies have identified a strong link between the gut microbiota and AS (Wang et al., 2011; Koeth et al., 2013; Jonsson and Backhed, 2017; Ma and Li, 2018), Trimethylamine N-oxide (TMAO), an important gut microbe-dependent metabolite, is generated from dietary choline, betaine and L-carnitine which are metabolized to trimethylamine (TMA) through gut microbiota metabolism and further converted to TMAO by hepatic flavin monooxygenases (FMOs) (Lang et al., 1998; Wang et al., 2011; Koeth et al., 2013; Jonsson and Backhed, 2017; Ma and Li, 2018). The mechanism of TMAO participating in AS is still under further investigation. It is undeniable that AS is a chronic inflammatory disease, and inflammation is constantly induced throughout the course of the disease (Ross, 1999; Libby et al., 2002; Tuttolomondo et al., 2012). Studies showed that increased TMAO level induced the activation of NF-kappa B (NF-κB) pathway and increased the expression of pro-inflammatory genes including inflammatory cytokines, adhesion molecules and chemokines (Seldin et al., 2016; Ma et al., 2017). Oxidative stress and NLRP3 inflammasome activation could also be triggered by TMAO, whereat inflammatory cytokines such as IL-18 and IL-1β were increased released (Sun et al., 2016; Boini et al., 2017). In addition, TMAO contributed to platelet hyperreactivity and thrombosis (Zhu et al., 2016; Zhu et al., 2017). In human clinical studies, elevated TMAO levels were associated with increased risk of AS and CVD (Wang et al., 2011; Koeth et al., 2013; Stubbs et al., 2016). Moreover, prospective cohort studies have shown that increased plasma TMAO levels predicted an elevated risk of major adverse cardiovascular events (MACE) such as MI, stroke or death (Tang et al., 2013; Tang et al., 2014; Wang et al., 2014; Li et al., 2017; Li et al., 2019). This article reviews the relationship between gut microbe-dependent TMAO and AS from the perspective of the mechanism including inflammation, inflammation-related immunity, cholesterol metabolism, and atherothrombosis, and its potential for clinical prognostic and as therapeutic target.

## TMAO Metabolism

Choline, L-carnitine, betaine, and other choline-containing compounds are the major nutrient precursors of gut microbe-dependent TMAO, a pro-atherogenic metabolite (Wang et al., 2011; Liu et al., 2016). These precursors are present in the human diet and are metabolized to TMA by the gut microbiota and various enzymes (al-Waiz et al., 1992; Koeth et al., 2013). TMA can be absorbed in the intestines and delivered to the liver through the portal circulation, where it is converted to TMAO by hepatic FMOs (Lang et al., 1998; Bennett et al., 2013; Zhu et al., 2018). In addition, natural preformed TMAO is remarkably high in fish, which can be directly absorbed after consumption and then excreted in the urine (Landfald et al., 2017).

Diet plays a key role in the generation of TMAO. L-carnitine and choline are mainly present in animal-origin foods, such as meat (especially red meat), meat products, eggs, and shellfish, while betaine is found mostly in plants (Jonsson and Backhed, 2017; Janeiro et al., 2018). Dietary L-carnitine is abundant in red meat and chronic supplementation was shown to accelerate AS by altering the microbial composition and increasing the production of TMA and TMAO (Koeth et al., 2013; Ferguson, 2013). Data from a prospective follow-up of 84,136 women over 26 years indicated that high intakes of red meat significantly elevated risk of coronary heart disease (CHD) (Bernstein et al., 2010). In addition, chronic consumption of red meat increased TMAO levels produced from carnitine, but not choline, and decreased renal TMAO excretion, and plasma TMAO levels decreased within 4 weeks after discontinuation of red meat consumption (Wang et al., 2019). γ-Butyrobetaine (GBB), a metabolite of dietary L-carnitine, is involved in the transformation of L-carnitine to the pro-atherogenic metabolite TMAO (Koeth et al., 2014). Koeth et al. (2013) found that omnivorous human subjects had higher TMAO levels than vegans or vegetarians after chronic L-carnitine supplementation. Their recent research further indicated that both omnivores and vegans or vegetarians could rapidly convert carnitine to GBB, while the subsequent gut microbial-dependent conversion of GBB to TMA was diet induced, especially through omnivorous dietary patterns and chronic L-carnitine exposure (Koeth et al., 2019). However, association between dietary choline/betaine, which are essential nutrient for health (Zeisel and Da Costa, 2009; Ueland, 2011), and risk of incident CVD was not be supported in meta-analysis, and plasma choline/betaine levels predicted risk of future MACE only when TMAO was elevated (Wang et al., 2014; Meyer and Shea, 2017). In addition, a high-fat or western-like diet increased plasma TMAO in human and animal studies (Boutagy et al., 2015; Chen et al., 2017), whereas the Mediterranean diet (MD) showed beneficial effects (De Filippis et al., 2016; Pignanelli et al., 2018).

The gut microbiota is another crucial factor in the generation of TMAO (Wang et al., 2011; Koeth et al., 2013; Ma and Li, 2018), as it has been shown to be essential for converting dietary compounds into TMA in gnotobiotic mice and human studies (Wang et al., 2011; Tang and Hazen, 2014; Zeisel and Warrier, 2017), and changes in the gut microbiota have marked effects on TMAO levels. For example, patients with large-artery atherosclerotic stroke and transient ischemic attack displayed obvious intestinal dysbacteriosis and reduced blood TMAO levels (Yin et al., 2015). In C57BL/6J mice, chronic exposure to the fungicide propamocarb induced significant changes in the gut microbial community structures, resulting in a significant increase in TMA levels in the feces (Wu et al., 2018). The microbial capacity for generating TMA appears to be important for the development of AS. Nine strains that can produce TMA from choline *in vitro* have been identified in the human gut. Low levels colonization of TMA-producing bacteria resulted in a significant accumulation of plasma TMAO in germ-free mice (Romano et al., 2015). It was also shown that TMA and TMAO levels were initially higher and the numbers of aortic lesions were increased in choline diet-fed ApoE^−/−^ mice transplanted with microbiota from high TMAO-producing C57BL/6J strains than in mice transplanted with low TMAO-producing NZW/LacJ strains (Gregory et al., 2015).

The final step in TMAO formation is the oxidation of TMA, which is mediated by the flavin-containing monooxygenase (FMO) family members FMO1 and FMO3. FMO3 exhibited 10-fold higher specific activity than FMO1, and thus plays a major role in TMAO formation (Bennett et al., 2013). Gender is another important factor in the oxidation of TMA. In female mice, most TMA N-oxygenation was catalyzed by FMO3, and in both genders, 11–12% of the TMA was converted to TMAO by FMO1 (Veeravalli et al., 2018). FMO3 was significantly down-regulated by testosterone in mice, suggesting the mechanism why in both humans and mice, the expression of hepatic FMO3 was lower in males than in females (Bennett et al., 2013). Consistent with these results, sterile female mice colonized with TMA-producing bacterial strains had higher plasma TMAO levels and hepatic FMO3 activity than male mice (Romano et al., 2015). Furthermore, FMO3 is regulated by farnesoid X receptor (FXR), a nuclear receptor activated by bile acid (Lefebvre et al., 2009), and injection of FXR ligands induced the expression of FMO3 and the production of TMAO in mice (Bennett et al., 2013).

TMAO levels were significantly associated with body mass index (BMI) in healthy adults with different risk factors (Wang et al., 2016), and studies in humans and mice have shown that plasma TMAO levels increased with ageing (Wang et al., 2014; Li et al., 2017.

## Possible Role of TMAO in Promoting AS

**Figure 1:**
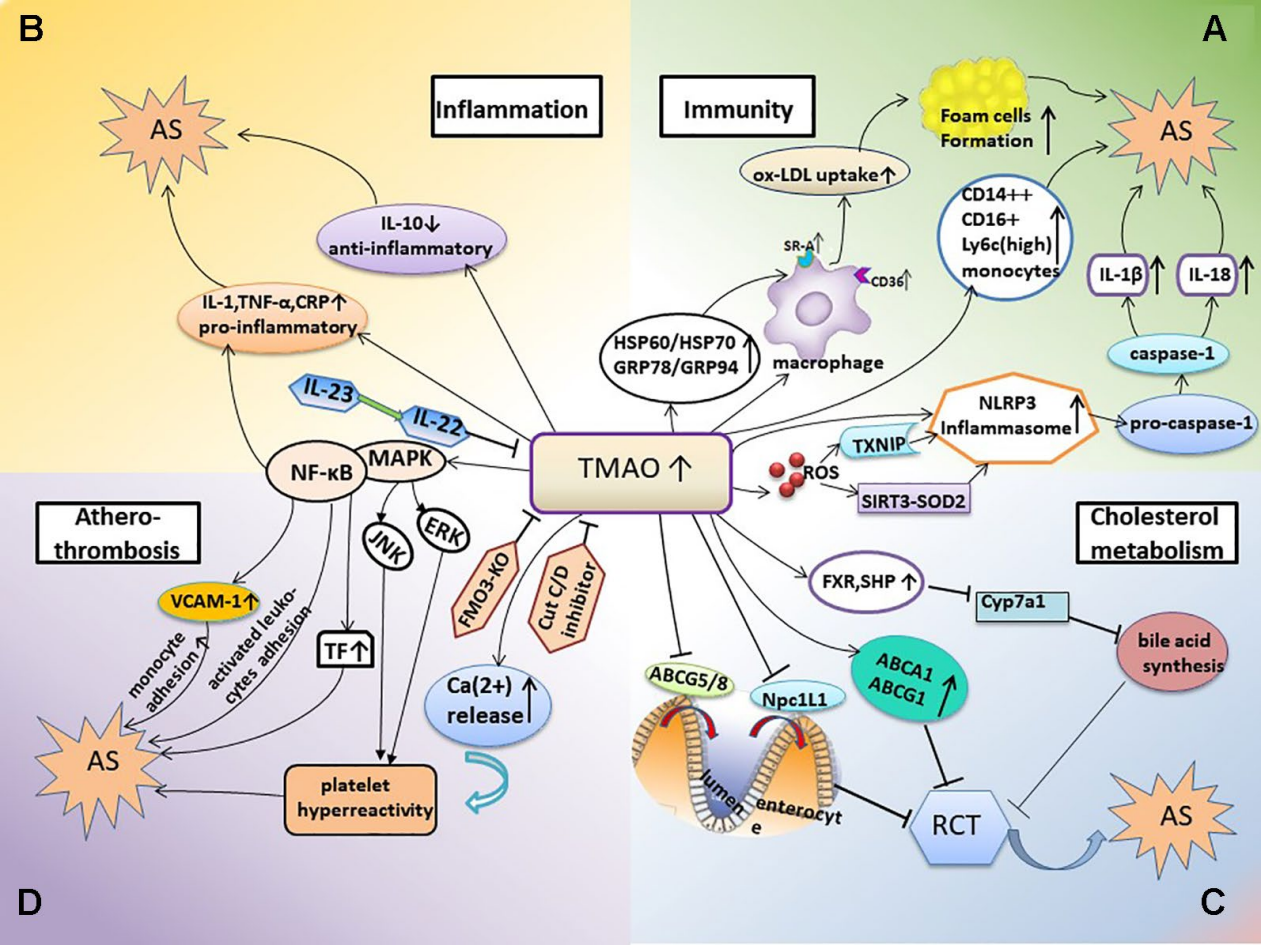
TMAO-involved mechanisms promoting AS. **(A)** TMAO and inflammation-immunity. In this mechanism, elevated TMAO activates the expression of SR-A1 and CD36 in macrophages, thus stimulating the uptake of ox-LDL and foam cell formation; the TMAO-induced increase in HSP expression is also involved in this process. TMAO levels are positively associated with monocyte activation and inflammation. Elevated TMAO levels also induce NLRP3 inflammasome activation and subsequently trigger inflammatory and immune responses. **(B)** TMAO and inflammation. Elevated TMAO levels lead to inflammation, accompanied with increased expression of pro-inflammatory cytokines and decreased expression of anti-inflammatory cytokines. **(C**, **D)** TMAO also inhibits bile acid synthesis and RCT, contributes to platelet hyperreactivity, and enhances the potential for thrombosis; all of which promote the occurrence of AS. AS, atherosclerosis; HSP, heat shock protein; RCT, reverse cholesterol transport; FXR, farnesoid X receptor; SHP, small heterodimer partner; Npc1L1, Niemann-Pick C1-like1; CRP, C-reactive protein; FMO3-KO, FMO-3 knockout; TF, tissue factor; VCAM-1, vascular cell adhesion molecule-1; JNK, c-JUN NH2-terminal protein kinase; ERK, extracellular signal-regulated kinase.

Below, we discuss the TMAO-involved mechanisms of AS from the perspectives of immunity, inflammation, cholesterol metabolism, and atherothrombosis (**Figure 1**).

### TMAO and Inflammation-Immunity

AS is a chronic inflammatory disease in which the innate and adaptive immune systems respond to many endogenous and exogenous molecules and highly diverse challenges (Ross, 1999; Belkaid and Hand, 2014). A large number of studies on the mechanism of AS have revealed that the net effect of immune activation is pro-atherogenic. Therefore, at least to some extent, AS should be considered as an autoimmune disease (Shoenfeld et al., 2001; Hansson, 2009). The role of TMAO in the immune mechanism of AS is summarized below (**Figure 1A**).

During the course of disease, autoantigen modification activates innate and adaptive immune responses. Heat shock protein (HSP) is an autoantigen found in atherosclerotic plaques and the blood circulation (Rodriguez-Iturbe and Johnson, 2018). Georg Wick et al. showed that the autoimmune reaction against HSP60 is the initial event of AS (Wick et al., 1995; Wick et al., 2004). Classical risk factors for AS trigger the cellular immune response in macrophages *via* the expression of stress-induced HSP (Wick et al., 2014). HSP shows a high degree of homology among species from microbes to humans (Khandia et al., 2017). Microbial or autologous HSP60 can be bound to endothelial cells *via* Toll-like receptors and serve as targets for autoimmune responses (Ohashi et al., 2000; Wick et al., 2004). Recent studies have demonstrated that gut microbe-dependent TMAO could induce changes in the expression or conformation of HSP. For example, studies performed in murine J774A.1 macrophages showed that TMAO induced stress and led to increased protein expression of GRP94 and HSP70, which may participate in the abnormal activation of macrophages involved in foam cell formation (Mohammadi et al., 2018). Similarly, TMAO increased the mRNA expression of the stress-induced heat shock proteins HSP60 and GRP78, a hallmark of endoplasmic reticulum stress induction, which was related to an increased risk of AS (Mohammadi et al., 2015).

The innate immune system recognizes the conserved structures of pathogens *via* a series of pattern recognition receptors (PRRs), prompting immune cells to respond and secrete inflammatory cytokines (Rock et al., 2010; Schroder and Tschopp, 2010; Takeuchi and Akira, 2010). Scavenger receptor (SRs), which include SR-A and CD36, are a group of typical PRRs on the surface of macrophages that can recognize and engulf oxidized low-density lipoprotein (ox-LDL) (Endemann et al., 1993; Boullier et al., 2001; Getz, 2005; Collot-Teixeira et al., 2007), another important autoantigen involved in AS (Maiolino et al., 2013). This engulfing is not regulated by the negative feedback of intracellular cholesterol. Therefore, lipid-overloaded macrophages turn into foam cells, which is one of the earliest cellular hallmarks of the atherosclerotic process (Bobryshev, 2006; Jin et al., 2018). TMAO activated the expression of SR-A1 and CD36 in macrophages, thus stimulating the uptake of ox-LDL and foam cell formation (Febbraio et al., 2000; Wang et al., 2011). When the production of TMAO was inhibited by antibiotics, the number of macrophages and the formation of foam cells in aortic lesions of ApoE^−/−^ mice were reduced (Wang et al., 2011). Other studies reported that TMAO increased the expression of CD36 and the formation of foam cells induced by ox-LDL, which could be reduced by siRNA-mediated knockdown of CD36 as well as inhibitors of MAPK (SB230580) and JNK (SP600125). This suggested that the CD36/MAPK/JNK pathway may play a key role in TMAO-induced foam cell formation (Geng et al., 2018). Targeted destruction of CD36 also prevented the development of atherosclerotic lesions in mice (Febbraio et al., 2000). However, *in vitro* study found that different concentrations of TMAO have no effect on the formation of foam cells in mouse macrophages (Collins et al., 2016).

The NLRP3 inflammasome is a polyprotein complex formed by the activation of PRRs that was recently reported to be crucial for the development of AS (Duewell et al., 2010; Hoseini et al., 2018). The activated NLRP3 inflammasome converted pro-caspase-1 to active caspase-1, which promoted the maturation and secretion of IL-18 and IL-1β and triggered the inflammatory and immune responses (Martinon et al., 2002; Martinon and Tschopp, 2007; Takahashi, 2014). Notably, studies have shown that elevated TMAO levels induce NLRP3 activation. For example, experiments using carotid artery endothelial cells (CAECs) and wild-type mice with partially ligated carotid arteries showed that TMAO significantly induced NLRP3 inflammasome activation and increased caspase-1 activity, IL-1β production, and cell permeability, which contributed to the endothelial injury that initiates AS (Koka et al., 2016; Boini et al., 2017). This mechanism may be related to both lysosomal dysfunction and redox regulation (Boini et al., 2017). Moreover, the essential of intracellular ROS in activating NLRP3 inflammatory was verified in hyperhomocysteinemia (HHcy) mice model (Wang et al., 2017). Activation of the TMAO-induced endothelial NLRP3 inflammasome was reduced by a mitochondrial ROS scavenger or SIRT3 overexpression in human umbilical vein endothelial cells (HUVECs), which suggested that the activation was mediated in part by inhibition of the SIRT3-SOD2-mtROS signaling pathway (Chen et al., 2017). Other studies proposed that this activation was mediated by the ROS-TXNIP pathway (Zhou et al., 2010; Sho and Xu, 2019). TXNIP is the most widely studied protein which links ROS and NLRP3 inflammasome (Zhou et al., 2010; Lane et al., 2013; Ye et al., 2017). Oxidative stress and activation of the TXNIP-NLRP3 inflammasome were triggered by TMAO, whereat inflammatory cytokines IL-18 and IL-1β were released in a dose- and time-dependent manner (Sun et al., 2016). In addition, studies performed in fetal human colon cells (FHCs) showed that TMAO caused dose- and time-dependent increases in NLRP3 inflammasome activation and ROS production (Yue et al., 2017).

TMAO levels were significantly correlated with the percentage of pro-inflammatory intermediate CD14^++^CD16^+^ monocytes in ischemic stroke patients (Haghikia et al., 2018). TMAO was also positively correlated with two biomarkers of monocyte activation and inflammation (sCD14 and sCD163) in HIV patients with carotid AS (Shan et al., 2018). More specifically, sCD14 was independently associated with TMAO in untreated HIV-infected subjects (Haissman et al., 2017). Moreover, the numbers of pro-inflammatory murine Ly6C^high^ monocytes were higher in mice fed a choline-rich diet which increased TMAO synthesis than in chow-fed control mice (Haghikia et al., 2018).

### TMAO and Inflammation

AS is a chronic inflammatory disease, and inflammation is constantly induced throughout the course of the disease (Ross, 1999; Libby et al., 2002; Tuttolomondo et al., 2012). Several studies have shown increased expression of pro-inflammatory cytokines when plasma TMAO levels were increased. For example, obese mice induced by feeding a western diet, which is a risk factor for AS (Boutagy et al., 2015; Chen et al., 2017), had higher plasma TMAO levels as well as increased expression of pro-inflammatory cytokines, including TNF-α and IL-1β, and decreased expression of the anti-inflammatory cytokine IL-10 (Chen et al., 2017) (**Figure 1B**). Moreover, a study of 271 German adults (Rohrmann et al., 2016) revealed a positive association between the plasma concentration of TMAO and low-grade inflammation. Subjects with elevated plasma TMAO levels had higher plasma levels of TNF-α, sTNF-R p75, and sTNF-R p55; however, there were no differences in the plasma levels of IL-6 and C-reactive protein (CRP). Another study suggested that TMAO levels were positively associated with IL-1β and hsCRP levels in 81 patients with stable angina (Chou et al., 2019). CRP is an inflammatory biomarker that is best validated currently and levels of CRP prospectively assess the risk of AS and atherosclerotic complications and CV risk stratification (Burke et al., 2002; Libby et al., 2002; Libby and Ridker, 2004). Therefore, the relationship between TMAO and CRP needs to be further determined. *In vitro* studies performed in cultured endothelial progenitor cells (EPCs) showed that TMAO promoted cellular inflammation and increased oxidative stress (Chou et al., 2019).

The NF-κB pathway plays a regulatory role in the expression of many AS-related pro-inflammatory genes (Tak and Firestein, 2001; Baker et al., 2011) (**Figure 1B**). Seldin et al. (2016) demonstrated that TMAO enhanced the expression of inflammatory genes in the aortic endothelium and smooth muscle cells and promoted the adhesion of activated leukocytes to endothelial cells in Ldlr^−/−^ mice fed a choline-rich diet. Pharmacological inhibition suggested that activation of mitogen-activated protein kinase (MAPK) and NF-κB signaling were required for these processes. In addition, TMAO induced several pro-atherogenic inflammatory proteins including cyclooxygenase 2, E-selectin, IL-6 and intracellular adhesion molecule 1 by activating NF-κB (Seldin et al., 2016). NF-κB is also a crucial regulator of atherosclerotic thrombosis. Studies on HUVECs demonstrated that TMAO increased monocyte adhesion, which was partly attributed to upregulation of vascular cell adhesion molecule-1 (VCAM-1) expression by activated protein kinase C (PKC) and p-NF-κB (Ma et al., 2017). Moreover, TMAO increased the expression and activity of tissue factor (TF) *via* activation of the NF-κB signaling pathway in primary human coronary artery endothelial cells (HCAECs), thereby promoting atherothrombosis (Cheng et al., 2019).

Inactivation of the IL-23–IL-22 signal led to a systemic increase of TMAO levels. Fatkhullina et al. (2018) proposed that IL-23 and its downstream target IL-22 repressed AS by inhibiting pro-atherogenic microbiota and microbial metabolites such as TMAO. IL-22 is a characteristic cytokine of Th17 myriad and IL-23 controls the function of Th17 subsets (Buonocore et al., 2010; Peshkova et al., 2016). Though Wang et al. revealed that IL-22 and TH17 pathway could alleviate metabolic disorders in diabetes (Wang et al., 2014), the role of IL-22 and IL-23 in AS remains to be determined.

### TMAO and Cholesterol Metabolism

TMAO plays a key regulatory role in lipid metabolism (**Figure 1C**). Studies in mice revealed that dietary supplementation with TMAO, choline, or carnitine decreased reverse cholesterol transport (RCT) (Janeiro et al., 2018), a mechanism that counteracts excess cholesterol deposition in peripheral tissues by transporting excess cholesterol to the liver and small intestine (Chistiakov et al., 2015). Ross and Glomset first proposed that atherosclerotic lesions developed when there was an imbalance between arterial cholesterol deposition and removal after endothelial injury, suggesting a relationship between RCT and AS (Glomset, 1968; Ross and Glomset, 1973).The major pathway for cholesterol elimination is the metabolic synthesis of bile acids in the liver (Zarate et al., 2016), and it was reported that the size of the total bile acid pool was significantly smaller in mice administered TMAO (Koeth et al., 2013). Other studies in ApoE^−/−^ mice demonstrated that TMAO repressed hepatic bile acid synthesis by inhibiting Cyp7a1 expression in the classical pathway of bile acid synthesis and that this inhibition might be mediated by activation of the nuclear receptor FXR and small heterodimer partner (SHP), thus accelerating the formation of aortic AS (Makishima et al., 1999; Ding et al., 2018). In peritoneal macrophages derived from C57BL/6J mice that were exposed to TMAO *in vitro*, the expression of ABCG1 and ABCA1 was modestly increased and cholesterol efflux was detected. Moreover, dietary supplementation with TMAO significantly decreased the expression of both enteral cholesterol transporters Niemann-Pick C1-like1 (Npc1L1), which transports cholesterol into enterocytes from the intestinal lumen, and ABCG5/8, which transports cholesterol out of enterocytes into the gut lumen (Jia et al., 2011; Koeth et al., 2013). However, it is not clear whether the changes in these transporters are involved in the observed systemic decrease in RCT induced by TMAO.

The TMA/FMO3/TMAO pathway driven by gut microbiota is also an important regulator of lipid metabolism (Bennett et al., 2013; Warrier et al., 2015). The TMAO-generating enzyme FMO3 reduced RCT, decreased the intestinal absorption of cholesterol, and changed the composition and size of the bile acid pool (Warrier et al., 2015; Al-Rubaye et al., 2019). Previous studies showed that mice lacking LXR were unable to induce the transcription of gene encoding Cyp7a, confirming the important role of LXR in dietary cholesterol metabolism (Peet et al., 1998; Chiang et al., 2001). In cholesterol-fed mice, knockdown of FMO3 induced macrophage RCT stimulated by liver X receptor (LXR), thus improving the cholesterol balance and protecting against AS (Warrier et al., 2015).

### TMAO and Atherosclerotic Thrombosis

In animal models, TMAO has been shown to directly cause AS and thrombosis (Koeth et al., 2013; Chen et al., 2016). The risk of platelet hyperreactivity and thrombosis is increased in many conditions associated with atherosclerotic CVD, such as oxidative stress and hyperlipidemia (Podrez et al., 2007; Chen et al., 2008). Evidence from human and animal model studies suggested one possible mechanism being that TMAO contributed to platelet hyperreactivity and enhanced the potential for thrombosis (Zhu et al., 2016; Zhu et al., 2017) (**Figure 1D**), which were correlated with prospective risk of coronary events and death and the extent of terminal organ injury such as myocardial injury (Trip et al., 1990; Kabbani et al., 2001; Kabbani et al., 2003; Frossard et al., 2004). Direct exposure of platelets to TMAO increased stimulus-dependent activation of platelets *via* multiple agonists by increasing the release of intracellular Ca^2+^ (Zhu et al., 2016). In addition, in pathological conditions of AS and hyperlipidemia, oxLDL activated platelets through a specific CD36-dependent platelet signaling pathway, in which increased activation of MAPK JNK2 and MAPK ERK5 were regard as critical mediators (Chen et al., 2008; Yang et al., 2017; Yang et al., 2018). Studies have confirmed that TMAO plays an important role in inducing foam cell formation and vascular inflammation by up-regulating the MAPK/JNK pathway and MAPK/ERK pathway (Geng et al., 2018; Wu et al., 2019). However, contrary to studies in the general populations, no evidence for TMAO-induced platelet hyperreactivity was detected in HIV-infected individuals (Haissman et al., 2017). Furthermore, as mentioned above, TMAO promoted atherothrombosis by increasing TF expression (Cheng et al., 2019), monocyte adhesion (Ma et al., 2017), and endothelial cell adhesion by activated leukocytes (Seldin et al., 2016), which were mediated by activation of the NF-κB signaling pathway.

Studies of microbial colonization in sterile mice have shown that increased platelet reactivity and thrombosis potential were sufficient to be delivered *via* microbial CutC-dependent TMA/TMAO production in a host (Skye et al., 2018) (**Figure 1D**). Consistently, oral administration of a CutC/D inhibitor markedly reduced plasma TMAO levels and rescued diet-induced platelet hyperreactivity and thrombosis without significant toxicity or increased bleeding risk (Roberts et al., 2018). Moreover, FMO3-knockout mice showed markedly reduced systemic TMAO levels and thrombosis potential (Shih et al., 2019). Recent studies proposed reducing platelet aggregation and arterial thrombosis *via* targeted suppression of gut microbial proteins associated with TMAO production as a promising therapeutic target (van Mens et al., 2019).

## Utility of TMAO for Predicting Clinical Risk and Prognostic Stratification in AS

TMAO may be a novel predictive biomarker for AS (**Table 1**). It was identified strongly associated with AS in a large independent clinical cohort for CVD (N = 1,876) (Wang et al., 2011). In CHD cohorts, Zhong et al. (2019) found that the plasma concentrations of TMAO, creatinine, choline, and carnitine were notably higher in CHD patients (n = 302) than in those with normal coronary arteries (n = 53). In addition, plasma TMAO was an independent predictor in CHD patients (n = 423) with or without type 2 diabetes mellitus (T2DM) (Dong et al., 2018). It was shown that urinary TMAO, but not its precursors, was correlated with the risk of CHD (n = 275) and may accelerate the development of CHD (Yu et al., 2019). In patients with ST-segment elevation myocardial infarction (STEMI; n = 335), elevated plasma TMAO levels predicted both a high SYNTAX score and the presence of multivessel disease, which were used to quantify the coronary atherosclerotic burden. Thus TMAO was associated with higher coronary atherosclerotic load in STEMI patients (Sheng et al., 2019). In HIV-infected individuals (n = 520), plasma TMAO levels were related to carotid AS progression, and higher TMAO levels were correlated with an enhanced risk of carotid plaques (Shan et al., 2018). Randrianarisoa et al. (2016) reported that serum TMAO levels had positive correlation with carotid intima-media thickness (cIMT), independent of established cardiovascular (CV) risk markers. However, there was no significant association between changes in TMAO levels and changes in cIMT across the population over 10-year follow-up (Meyer et al., 2016). In addition, TMAO levels was similar in subjects with or without carotid atherosclerotic plaques (Yin et al., 2015). TMAO may not significantly promote the risk of early atherosclerotic disease in healthy adults (Meyer et al., 2016).

**Table 1:** Human studies of TMAO as a potential novel and independent risk factor for predicting clinical risk of atherosclerosis (AS) and prognostic stratification.

	Study	Patient population	Main findings/outcomes
Positive results	Wang et al., 2011	Subjects undergoing selective cardiac evaluations (N = 1,876)	Elevated levels of fasting choline, TMAO and betaine were dose-dependent associated with the risk of CVD
	(Zhong et al., 2019)	302 with CHD and 59 with NCA in southern China	Plasma concentrations of TMAO, creatinine, choline, and carnitine were notably higher in CHD patients than in those with NCA
	(Dong et al., 2018)	132 controls, 243 with CHD, and 175 with CHD and T2DMv	Plasma TMAO levels were remarkably higher in CHD patients than in controls and were significantly elevated in CHD patients with T2DM; TMAO was an independent predictor in CHD patients with or without T2DM
	(Yu et al., 2019)	275 with CHD and 275 controls	Urinary TMAO, but not its precursors, was correlated with a risk of CHD and may accelerate the development of CHD
	(Sheng et al., 2019)	335 with STEMI and 53 healthy controls	TMAO levels were higher in STEMI; elevated plasma TMAO levels predicted both a high SYNTAX score and the presence of multivessel disease and were associated with higher coronary atherosclerotic load
	(Shan et al., 2018)	520 HIV-infected and 217 uninfected (112 incident plaque cases)	In HIV-infected individuals, higher TMAO levels were correlated with an enhanced risk of carotid plaques
	(Randrianarisoa et al., 2016)	220 subjects in the Tübingen lifestyle intervention program	Newly demonstrated that elevated serum TMAO levels had positive correlation with increased cIMT
	Tang et al., 2013	4007 patients undergoing elective coronary angiography	Elevated plasma TMAO levels were associated with an increased risk of incident MACE.
	(Li et al., 2019)	530 with chest pain (suspected ACS) and 1683 with ACS	Elevated TMAO/TML levels were correlated with MACE over both 30 days and 6 months of follow-up and were also relevant to incident long-term (1-year and 7-year) all-cause mortality
	Tang et al., 2014	720 patients with stable heart failure	Elevated plasma TMAO levels were associated with a 3.4-fold enhanced mortality risk and predicted 5-year mortality risk.
	(Senthong et al., 2016)	2235 with stable CAD; 935 with PAD	Higher plasma TMAO levels were respectively associated with a 4-fold and 2.7-fold enhanced mortality risk in a 5-year follow-up period and could predict 5-year all-cause mortality risk.
	(Suzuki et al., 2017)	1079 with acute MI	TMAO independently predicted death/MI at 2 years, but was not able to predict death/MI at 6 months, and was superior to currently used biomarkers
	(Haghikia et al., 2018)	78 and 593 with recent prior ischemic stroke	Both cohorts showed that higher plasma TMAO levels were related to an increased risk of subsequent CV events
Negative results	Yin et al., 2015	322 patients with atherosclerotic ischemic stroke and TIA and 231 asymptomatic AS controls	Stroke and TIA patients had significantly lower TMAO levels than asymptomatic group, rather than higher. And there was no significant change in blood TMAO levels in asymptomatic atherosclerotic controls.
	Meyer et al., 2016	817 participants	TMAO was not associated with cIMT, a measure of AS, during10-year follow-up.
	(Skagen et al., 2016)	264 with carotid artery AS and 62 healthy controls	No remarkable association between TMAO and CV mortality was found
	Kaysen et al., 2015	235 patients receiving hemodialysis	No obvious association between serum TMAO levels and hospitalizations or CV death and all-cause mortality.
	Mueller et al., 2015	339 patients of suspected CAD.	Plasma TMAO or betaine levels were not associated with the presence of CHD or MI history or incident CV events during 8-year follow-up.

CHD, coronary heart disease; NCA, normal coronary arteries; T2DM, type 2 diabetes mellitus; STEMI, ST-segment elevation myocardial infarction; MI, myocardial infarction; cIMT, carotid intima-media thickness; MACE, major adverse cardiovascular events; ACS, acute coronary syndromes; CAD, coronary artery disease; PAD, peripheral artery disease; TIA, transient ischemic attack.

Prospective cohort studies have shown that increased plasma TMAO levels predicted an elevated risk of MACE in patients with pre-existing AS (**Table 1**). A 3-year follow-up of 4,007 patients undergoing elective coronary angiography revealed a significant association between elevated plasma TMAO levels and increased risk of MACE (stroke, myocardial infarction (MI), or death) (Tang et al., 2013). In patients with chest pain (n = 530) and acute coronary syndromes (ACS) (n = 1683), plasma levels of TMAO and its nutrient precursor TML could predict both the near-term (30-day/6-month) and long-term (1–7-year) risks of CV events (Li et al., 2017; Li et al., 2019). Among patients with stable coronary artery disease (n = 2235) (Senthong et al., 2016), heart failure (n = 720) (Tang et al., 2014) and peripheral artery disease (PAD) (n = 935) (Senthong et al., 2016), elevated TMAO levels could predict 5-year mortality and increased plasma TMAO levels were respectively associated with a 4-fold, 3.4-fold and 2.7-fold increased mortality risk. Moreover, TMAO independently predicted all-cause mortality or reinfarction (death/MI) at 2 years but was not able to predict death/MI at 6 months in patients hospitalized for acute MI (n = 1079). However, TMAO improved risk stratification for death/MI at 6 months by down-classifying the risk of some patients (Suzuki et al., 2017). Another study also demonstrated a graded relationship between plasma TMAO levels and the risk of subsequent CV events among patients presenting with recent prior ischemic stroke (Haghikia et al., 2018). In addition, higher plasma L-carnitine, choline and betaine levels portended increased risks for CVD and incident MACE, but only among individuals with high TMAO levels simultaneously (Koeth et al., 2013; Wang et al., 2014). However this association was not be supported in meta-analysis (Meyer and Shea, 2017). Furthermore, several studies also showed no remarkable association between TMAO and history of CHD or incident CV events or CV mortality (Kaysen et al., 2015; Mueller et al., 2015; Skagen et al., 2016).

In summary, current researches suggest that TMAO levels could be potentially combined with existing risk stratification tools and may offer a novel approach for the prevention and treatment of atherosclerotic disease. Further studies are needed to define the plasma TMAO levels that represent increased risk and establish the correlation between metabolite concentrations and increased CV/MACE risk.

## Potential of TMAO as a Therapeutic Target in AS

**Figure 2:**
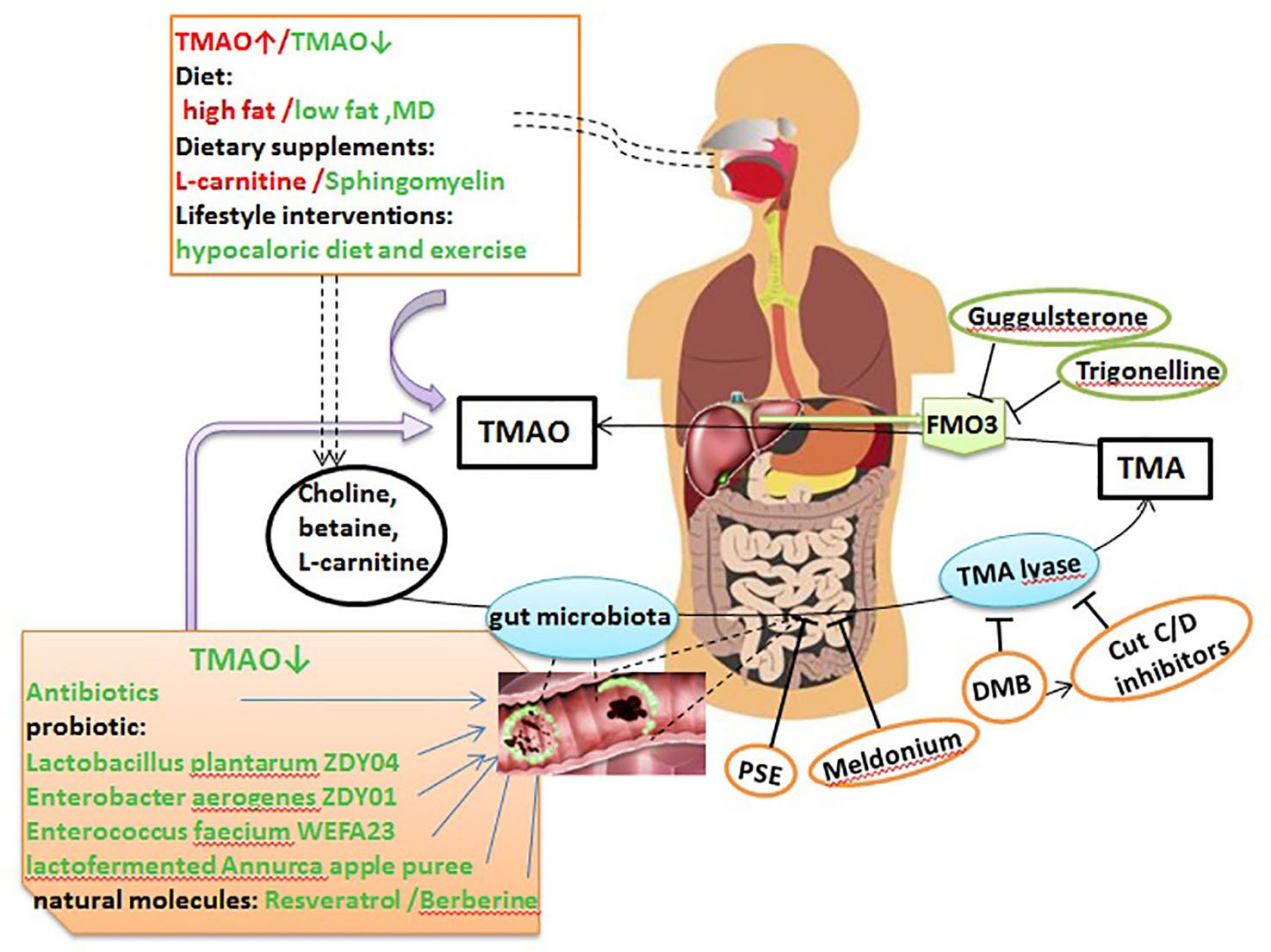
TMAO potential as a therapeutic target in AS. Dietary choline, L-carnitine, betaine, and other choline-containing compounds are the major nutrient precursors of TMAO, and they are metabolized to TMA by the gut microbiota and various enzymes. Then, TMA can be absorbed in the intestines and delivered to the liver through the portal circulation, where it is converted to TMAO by FMO3. TMAO can be targeted as follows: *via* the diet, dietary supplements and lifestyle interventions can significantly affect TMAO levels; antibiotics, probiotics, probiotic functional products, and some natural molecules can markedly decrease TMA and TMAO levels by remodeling the gut microbiota; PSE, meldonium, DMB, and CutC/D inhibitors can suppress the generation of TMA; and trigonelline and guggulsterone can inhibit the conversion of TMA to TMAO by inhibiting FMO3. MD, Mediterranean diet; DMB, 3,3-dimethyl-1-butanol; PSE, plant sterol ester.

Several therapeutic strategies to lower TMAO levels that are currently being explored are summarized below (**Figure 2**).

### Dietary Control of TMAO and AS

Diet is an important factor affecting TMAO levels and the progression of AS (Bennett et al., 2013). Elevated TMAO levels were observed over a 4-week interval in individuals consuming a high-fat diet (HFD) that is predominantly animal based, compared to individuals consuming a low fat and the MD (Boutagy et al., 2015; Park et al., 2019), but there was no difference in fasting TMAO levels during a 2-week HFD intervention (Boutagy et al., 2015). The MD, a nearly vegetarian diet, was proved to be the strongest evidence for the dietary prevention of major CV events (De Lorgeril et al., 1999; Estruch et al., 2013; Spence, 2018). Greater adherence to MD showed a beneficial role on reducing TMAO levels, CV and overall mortality (Trichopoulou et al., 2003; Sofi et al., 2008; De Filippis et al., 2016). It is noteworthy that gender has a significant influence on MD adherence. In 144 healthy adults with MD, males presented lower adherence to the MD, higher energy intake and higher TMAO levels than females (Barrea et al., 2019). However, another study found no significant changes in fasting TMAO levels after a 6-month intervention with the MD in 115 healthy adults with an elevated risk of colon cancer (Griffin et al., 2019). These findings showed that fasting TMAO levels are not strongly regulated by a moderate modification of the diet (Randrianarisoa et al., 2016).

Lifestyle interventions can also affect plasma and urine levels of TMAO. For example, among 16 obese adults, compared to a eucaloric diet and exercise, a 12-week hypocaloric diet and exercise decreased the percent change in TMAO (Erickson et al., 2019). Moreover, TMAO levels were reduced in 34 prepubertal obese children after a 6-month lifestyle intervention; notably, changes in TMAO concentrations after intervention were not related to choline intake but were negatively correlated with fiber intake (Leal-Witt et al., 2018). However, another study confirmed that the mean TMAO levels did not change during the lifestyle intervention (Randrianarisoa et al., 2016). Recent studies have demonstrated the effect of dietary supplements on TMAO and AS. Samulak et al. (2019) reported that oral L-carnitine supplementation markedly elevated plasma TMAO levels, but did not induce variations in the lipid profile or other adverse CV event markers in healthy older women within 24 weeks. Dietary sphingomyelin (SM) supplementation also remarkably decreased the atherosclerotic lesion area in the aortic arch in chow-fed ApoE^−/−^ mice, reduced serum TMAO levels in C57BL/6 mice, and did not affect circulating SM levels or increase AS in ApoE^−/−^ mice fed a HFD (Chung et al., 2017).

### Regulating the Gut Microbiota to Reduce TMAO Levels

Evidence from experimental and clinical studies has confirmed the role of the gut microbiota in TMAO metabolism, providing a theoretical basis for regulating the gut microbiota to control TMAO levels and prevent or treat AS (Wang et al., 2011; Tang and Hazen, 2014). The use of broad-spectrum antibiotics is the easiest method to alter the gut microbiota and thereby regulate TMAO levels. In healthy participants, plasma TMAO levels were increased in a time-dependent manner after phosphatidylcholine challenge; these levels were significantly reduced after administration of antibiotics, and then increased after discontinuation of antibiotic administration (Tang et al., 2013). In animal experiments, choline supplementation increased AS nearly 3-fold in male and female mice without antibiotics. In contrast, suppression of the gut microbiota with antibiotics completely inhibited this dietary choline-mediated enhancement in AS (Wang et al., 2011). Ageing also altered the abundance of the intestinal flora, and plasma TMAO levels increased accordingly. Inhibition of the gut microbiota by the addition of broad-spectrum antibiotics to drinking water for 3–4 weeks ameliorated age-related oxidative stress and arterial dysfunction in mice (Brunt et al., 2019). However, it should be noted that the chronic use of antibiotics may have adverse consequences, such as the emergence of antibiotic-resistant bacterial strains and inducing insulin resistance and obesity (Cho et al., 2012; Tang and Hazen, 2014). Thus, additional studies are needed to explore its safety.

The use of probiotics, probiotics and synbiotics, and probiotic functional products is a safer and potentially more effective way to alter the microbiota composition. In animal experiments, administration of the probiotic strains *Lactobacillus plantarum* ZDY04 (Qiu et al., 2018) and *Enterobacter aerogenes* ZDY01 (Qiu et al., 2017) markedly decreased choline-induced cecal TMA and serum TMAO levels by remodeling the gut microbiota in mice. Gut colonization with methanogenic archaea reduced plasma TMAO levels and attenuated the burden of AS, with decreased area and fat content in the atherosclerotic plaques, in Apo E^−/−^ mice fed a TMA-supplemented or high choline diet (Ramezani et al., 2018). *Enterococcus faecium* WEFA23 improved the diversity of the gut microbiota in rats fed a HFD and decreased TMAO production and cholesterol levels (Huang et al., 2018). In human clinical trials, a study of 90 individuals with atherosclerotic CV disease risk factors suggested that Lactofermented Annurca apple puree was an effective functional food that could effectively control plasma TMAO and HDL-C levels (Tenore et al., 2019). Unfortunately, treatment with the multi-strain probiotic VSL#3 in non-obese males (Boutagy et al., 2015) and the probiotic strain *Lactobacillus casei* Shirota in patients with metabolic syndrome did not reduce the increased fasting plasma TMAO levels after a HFD (Tripolt et al., 2015). In general, probiotics that have been shown to effectively lower TMAO levels in human studies are relatively scarce.

Some natural molecules play a protective role against AS, primarily by remodeling the intestinal microbiota. Resveratrol (RSV), a natural plant antitoxin with probiotic activity, was found to attenuate TMAO-induced AS by lowering TMAO levels and increasing hepatic bile acid synthesis by remodeling the gut microbiota (Chen et al., 2016). Berberine (BBR) has been shown to have antimicrobial effects; in BBR-treated male ApoE^−/−^ mice fed a HFD, the abundances of *Firmicutes* and *Verrucomicrobia* were changed and the expression of hepatic FMO3 and serum TMAO levels were markedly reduced (Shi et al., 2018).

### Inhibition of TMA Generation

Choline TMA-lyase (CutC/CutD) and carnitine oxygenase (CntA) are several enzymes that involved in converting dietary compounds into TMA. Rath et al. (Rath et al., 2017) examined the TMA-forming potential of microbial communities and found that *cutC* amplicons were related to various taxa, but that the sequences showed low nucleotide identities to reference sequences, whereas *cntA* amplicons showed high identities to reference sequences, principally sequences from *Escherichia coli*. This provided critical information for the development of particular treatment strategies that inhibit TMA producers. Wang et al. (2015) demonstrated for the first time that 3,3-dimethyl-1-butanol (DMB), a structural analogue of choline, could non-lethally inhibit TMA formation by inhibiting distinct microbial TMA lyases and thus prevent the development of atherosclerotic lesions in ApoE^−/−^ mice. They further modified DMB and developed inhibitors targeting CutC and CutD, the major microbial TMA-generating enzyme pair, and when administered as a single oral dose, significantly decreased plasma TMAO concentrations for up to 3 days and rescued the diet-induced enhancements in platelet reactivity and thrombosis in animal models, without increasing bleeding risk or toxicity (Roberts et al., 2018). In addition, DMB significantly attenuated but did not completely eliminate pulmonary artery AS induced by an 8-week exposure to intermittent hypoxia and hypercapnia (IHC) in ApoE^−/−^ mice and Ldlr^−/−^ mice (Xue et al., 2017). Elevated circulating TMAO levels resulted in endothelial dysfunction in elderly rats, which was reversed by DMB treatment for 8 weeks (Li et al., 2017). Plant sterol esters (PSEs) were also shown to markedly dampen microbial production of TMA, attenuate cholesterol accumulation, and nearly abolish atherogenesis in ApoE^−/−^ mice (Ryan et al., 2017). GBB is a pro-atherogenic intermediate in the conversion of L-carnitine to TMAO controlled by the gut microbiota (Koeth et al., 2014). Meldonium, an analogue of GBB, significantly decreased gut microbiota-dependent TMA/TMAO production from L-carnitine in Wistar rats (Kuka et al., 2014). These results indicate that targeting gut microbial TMA production and the use of non-microbicidal inhibitors are potential therapeutics for AS.

### Inhibition of TMA–TMAO Conversion

FMO3 is a critical enzyme in the conversion of TMA to TMAO. It also plays an important role in modulating glucose and lipid homeostasis (Warrier et al., 2015; Shih et al., 2015; Shih et al., 2015), and knockdown or silence of hepatic FMO3 in different mouse strains reduced plasma TMAO levels, altered cholesterol and lipid metabolism, and decreased AS (Bennett et al., 2013; Warrier et al., 2015; Miao et al., 2015; Schugar and Brown, 2015). Furthermore, FMO3 suppression and overexpression were shown to directly impact systemic TMAO levels, platelet reactivity, and thrombosis rates in a murine model of FeCl_3_-induced carotid artery injury (Zhu et al., 2018). A recent study showed that trigonelline, a compound from *Trigonella foenum-graecum*, inhibited the conversion of TMA to TMAO by inhibiting FMO3. In additon, culturing *Citrobacter freundii* in choline-enriched medium supplemented with trigonelline led to significant reductions in TMA and subsequent TMAO production. In an ex vivo study, TMAO production was reduced by a maximum of 85.3% in the presence of 300 µg/mL trigonelline (Anwar et al., 2018). Furthermore, activation of the nuclear receptor FXR induced the expression of FMO3 and the production of TMAO (Bennett et al., 2013). Recent evidence from *in vitro* and *in vivo* studies showed that guggulsterone, a FXR antagonist, lowered plasma TMA/TMAO levels (Gautam et al., 2018). However, inhibition of FMO3 expression led to a large accumulation of TMA, resulting in trimethylaminuria, which is better known as “fish odor syndrome,” and seriously affected patient quality of life (Treacy et al., 1998; Messenger et al., 2013). In addition, since FMO3 plays a systemic role in catecholamine metabolism, inhibiting its function may not be innocuous (DiNicolantonio and McCarty M, 2019).

## Conclusions

AS is a complex disease, which makes characterizing its underlying mechanisms difficult. However, increasing evidence suggests a correlation between TMAO levels and the risk of AS. TMAO might influence AS by activating immune and inflammatory responses, altering cholesterol metabolism, and promoting atherosclerotic thrombosis (**Figure 1**). In addition, elevated TMAO levels are related to an increased risk of incident MACE among patients presenting with AS; thus, the addition of TMAO levels could improve existing risk stratification tools. Finally, TMAO could be used as a novel approach for the prevention and treatment of AS. Current studies have demonstrated that inhibiting various steps of TMAO production can reduce TMAO levels and treat AS (**Figure 2**). However, it is worth noting that inhibition of TMAO may also have adverse effects. We hope that novel TMAO-targeting therapeutic strategies for AS will be established in the near future.

## Author Contributions

YX and MW designed the manuscript. SY wrote the manuscript. LL revised the manuscript. XinL and FY searched the literature. RZ, XP, JL, LT, and XiaL aided in the design of the illustrations. All authors approved the manuscript for publication.

## Funding

The work was supported by the National Natural Science Foundation of China (Grant No. 81430098), the National Key R&D Program of China (2018YFC1704901) and the Beijing Natural Science Foundation (No.7172185).

## Conflict of Interest

The authors declare that the research was conducted in the absence of any commercial or financial relationships that could be construed as a potential conflict of interest.
